# The function of wing bullae in mayflies (Insecta: Ephemeroptera) reveals new insights into the early evolution of Pterygota

**DOI:** 10.1186/s12915-023-01750-8

**Published:** 2023-11-23

**Authors:** Eduardo Domínguez, Thomas van de Kamp, István Mikó, M. Gabriela Cuezzo, Arnold H. Staniczek

**Affiliations:** 1 Instituto de Biodiversidad Neotropical (IBN), CONICET- Facultad de Ciencias Naturales, Universidad Nacional de Tucumán (U.N.T.), Miguel Lillo 205, 4000 Tucumán, Argentina; 2 Institute for Photon Science and Synchrotron Radiation (IPS), Karlsruhe Institute of Technology (KIT), 76344 Eggenstein‐Leopoldshafen, Germany; 3 Laboratory for Applications of Synchrotron Radiation (LAS), Karlsruhe Institute of Technology (KIT), 76131 Karlsruhe, Germany; 4 Department of Biological Sciences, University of New Hampshire, Durham, NH 03824 USA; 5 Department of Entomology, State Museum of Natural History Stuttgart, Rosenstein 1, 70191 Stuttgart, Germany

**Keywords:** Bulla, Functional morphology, Flight mechanics, Insect flight, Subimago, Wing evolution, Ephemerida, Paleoptera

## Abstract

**Background:**

Mayflies are basal winged insects of crucial importance for the understanding of the early evolution of Pterygota. Unlike all other insects, they have two successive winged stages, the subimago and the imago. Their forewings feature so-called bullae, which are desclerotized spots in the anterior main veins. Up to now, they have been considered to play a major role in wing bending during flight.

**Results:**

We investigated bullae by multiple methods to reveal their structure and arrangement and to gain new information on the evolution of insect flight. Bullae are mostly present in the anterior negative wing veins, disrupting the otherwise rigid veins. High-speed videography reveals that mayfly wings do not bend during flight. Likewise, different arrangements of bullae in different species do not correlate with different modes of flying. Observations on the moulting of subimagines unravel that they are essential for wing bending during the extraction of the imaginal wing from the subimaginal cuticle. Bullae define predetermined bending lines, which, together with a highly flexible wing membrane enriched with resilin, permit wing bending during subimaginal moulting. Bullae are only absent in those species that remain in the subimaginal stage or that use modified modes of moulting. Bullae are also visible in fossil mayflies and can be traced back to stemgroup mayflies of the Early Permian, the 270 million years old Protereismatidae, which most probably had bullae in both fore- and hind wings.

**Conclusions:**

Bullae in mayfly wings do not play a role in flight as previously thought, but are crucial for wing bending during subimaginal moulting. Thus, the presence of bullae is a reliable morphological marker for a subimaginal life stage, confirming the existence of the subimago already in Permian Protereismatidae. A thorough search for bullae in fossils of other pterygote lineages may reveal wheather they also had subimagines and at what point in evolution this life stage was lost. In mayflies, however, the subimago may have been retained due to selective advantages in connection with the transition from aquatic to terrestrial life or due to morphological requirements for a specialized mating flight.

**Supplementary Information:**

The online version contains supplementary material available at 10.1186/s12915-023-01750-8.

## Background

Mayflies are one of the oldest branches of winged insects [1–4]. They are unique among pterygotes in having a penultimate winged life stage, the so-called subimago, which generally resembles the morphology of the adult [5]. Subimagines however differ from imagines in having duller colours, non-transparent wings covered with numerous microtrichia, shorter caudal filaments and legs (especially male forelegs), and yet non-functional genitalia. Only in a final second moult, which takes place within minutes to a few days after the transition from nymph to subimago, the mature imago will emerge to swarm and mate. The nuptial flight in adult mayflies generally takes places in the air, where male adults gather in swarms to perform a characteristic mating flight. It is usually directed in a more or less vertical direction with active wing strokes during ascending, followed by passive parachuting with spread wings, legs, and tail filaments during descent. Usually, the females fly into these male swarms, where the males approach them from below. The males of most species have drastically elongated forelegs in order to embrace the wing bases of the females from underneath during mating. Additionally, the males also grasp the female abdomen from below with their abdominal gonopodes (forceps) [6]. Within Ephemeroptera, there are different modes of swarming, which may involve horizontal swarming close to the water surface or even mating directly on it [7]. After aerial mating, the females would usually do a compensatory flight upstream to lay their eggs and to die shortly thereafter [8].

In the past, there have been many theories on the evolutionary origin and functional role of the subimago [5]. It has mostly been interpreted as a remnant of the moulting in adults, which still takes place in primarily wingless hexapod orders, i.e. all Entognatha, Archaeognatha, and Zygentoma [9, 10]. This interpretation seems straightforward, as wings in Paleozoic and Mesozoic stemgroup mayflies grew gradually as lateral outgrowths, involving numerous moults [11, 12]. However, while the retained subimaginal moulting in modern mayflies as such is certainly a plesiomorphic trait, some authors also have pointed out that there are most likely some selective advantages in the subimago. The coverage of subimaginal body and wings with numerous microtrichia has been assumed to have a hydrophobic effect, enabling the subimago to leave the water without wetting [5, 13]. However, the microsculptured cuticle of adult mayfly wings is most probably due to an epicuticular wax layer, which has hydrophobic properties [14, 15], so unwettability would not require a subimaginal stage per se. In a different approach to explain a pertaining subimaginal stage, Maiorana [16] pointed out the comparatively dramatic enlargement of male forelegs and tail filaments from nymph to imago in mayflies and reasoned that the subimaginal life stage between nymph to imago is simply needed as intermediate step to accomplish this multiple extension of the extremities. Recently, studies on the underlying genetic [17, 18] and hormonal mechanisms of metamorphosis [19] confirmed these previous interpretations of the phylogenetic and functional aspects of the subimago in mayflies.

Regardless of differences in setation and transparency, subimaginal and imaginal wings do resemble each other in size, shape, and venation. Mayflies are plesiomorphic in having highly corrugated wings with pronounced alternating positive and negative veins at different levels. Together with numerous cross veins between the main longitudinal veins, they provide for rigidity and stiffness of the wings [20].

Unlike other insect orders, mayfly wings are equipped with so-called bullae. These desclerotized, oval or rounded blister-like spots have so far been found mainly in some of the main negative, i.e. concave, longitudinal veins.

In the past, different authors applied the term “bulla” to different structures. Comstock [21], p.74] defined bullae in Hymenoptera as “weakened places in veins of the wing where they are crossed by furrows. The bullae are usually paler in colour than the other portions of the wing; they are common in the wings of Hymenoptera and some other insects.” On the opposite, Torre-Bueno [22], p.39] referred to the bulla as “a blister or blister-like structure; … in Ephemeridae, a slightly swollen part of the costal area of the wing toward the tip, with more crossveins, practically equivalent to the stigma, q.v.; which are weak spots on some of the wing veins where they are crossed by furrows (Comstock)”, thus referring to a wing region, which is today commonly referred to as pterostigma. In a revised edition of Torre-Bueno, Nichols [23] restricted the definition of bulla to the original use of Comstock, but several authors also have been using the term for the basal thickening of the main longitudinal veins throughout insects, often with emphasis on Palaeoptera [24].

In this work, we follow Edmunds & Traver [20], who applied the term to local, oval, widened blisters with lesser degree of sclerotization at approximately half-length of certain longitudinal veins in the forewings of subimagines and imagines of mayflies.

Traditionally, it has been suggested that wing bullae might play a central role in the flight of Ephemeroptera [20]. According to these authors, bullae form a line that would allow the distal part of the wing membrane to bend in the upstroke, diminishing the pressure and reducing the resistance. In the downstroke, the wing would not bend, getting a maximum of lift and propulsion. Wootton [25–27] described the longitudinal and transversal flexion lines in different insect wings and concluded that the latter are mainly related to the ventral bending of the wings during flight. Brodsky [7, 28] and Brodsky & Ivanov [29], investigated the thorax muscles and wing morphology of *Ephemera vulgata*. However, these authors rather postulated a gradual longitudinal flexion of the wing along the main convex veins during upstroke. Nevertheless, Brodsky [30] p. 94, Fig. 5.9a] also indicated two transversal flexion lines through the bullae that together would allow wing tip deflection in the same way that Edmunds & Traver [20] had proposed originally, which was adopted by Wootton [31]. He maintained that bullae are characteristic of those mayflies in which the males present a vertical nuptial flight and used paper models to study the physical designs of wings and to explain parallel adaptations in different insect groups [31]. For him, bullae would allow a compression of the veins, which would be necessary for the flattening of the wing during its upstroke bending. Wootton [31], based on his paper models and Brodsky’s [30] studies, adopted Edmunds’ & Traver’s [20] hypothesis and interpreted mayfly bullae as an adaptation for ventral wing bending during flight. When Grodnitsky [32] investigated the thyridium, a desclerotized spot in the wings of caddisflies, he traced a trajectory line between thyridium and arculus, the apical fusion of anal veins, along which the wings would bend. Domínguez & Abdala [33] also used trajectory lines when they studied size and location of bullae in leptophlebiid mayflies. Among their objectives was to prove if trajectory lines could be associated with certain types of flight within mayflies. However, they noted that—unlike predicted by Wootton [31]—there was no correspondence between presence or absence of bullae, trajectory lines, and a specific type of mating flight [33].

All the abovementioned inconsistencies in the descriptions of the flight in mayflies and the underlying morphological wing characteristics led to the present study.

## Results

### Wing movements during lift-off and flight

**Fig. 1:**
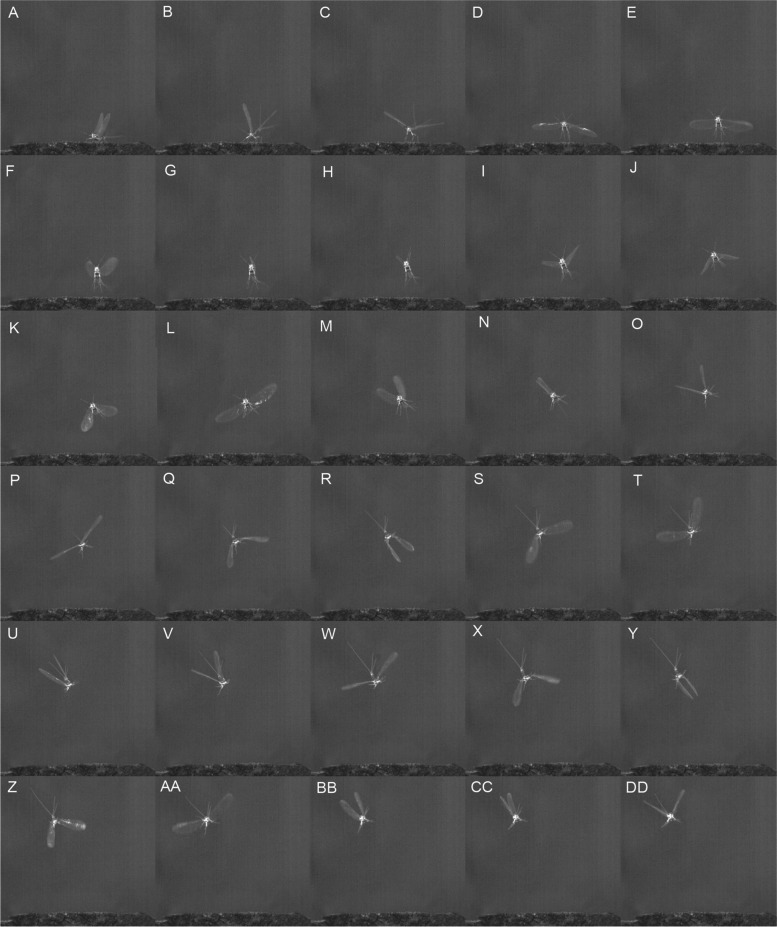
*Thraulodes cochunaensis* (Leptophlebiidae), still photographies from high-speed video S1 at 1677 frames per second showing first six wing cycles at lift-off. **A** Resting position, **B–D** initial jump-off and first downstroke, **E–G** first upstroke, **H–K** second downstroke, **L–N** third upstroke, **O–R** fourth downstroke, **S–U** fifth upstroke, **V–Y** fifth downstroke, **Z–BB** sixth upstroke, **CC–DD** sixth downstroke

Males and females of *Thraulodes cochunaensis* (Fig. 1, video S1) and *T. consortis* (Leptophlebiidae), *Leptohyphes eximius* (Leptohyphidae) (video S2) and *Callibaetis guttatus* (Baetidae) were high-speed recorded at lift-off and initial flight. We observed the same type of flight also in other families, though without recording them, e.g. in *Siphlonurus lacustris* (Siphlonuridae), *Epeorus assimilis* (Heptageniidae), *Ephemera danica* (Ephemeridae), *Serratella ignita* (Ephemerellidae), and *Caenis gonseri* (Caenidae). All recorded specimens essentially have identical patterns of wing cycles. In all four species, the hind wings are highly reduced and hardly visible at all, so we only consider the movement of the forewings in the following description of *T. cochunaensis* (Fig. 1): At rest, all wings are folded upwards and held vertically above the abdomen with the dorsal sides of the forewings touching each other. The anterior margin of the wing is thereby obliquely directed in a 45° angle in such a manner that the wing tip marks the highest and most posterior point of the forewing (Fig. 1A). The entire wing cycle resembles the typical movement of a rowing paddle, albeit in a vertical rather than horizontal movement. The lift-off is initiated by a firm push-off with all legs, which is accompanied by the first partial downstroke of the wings (Fig. 1B–D). This initial downstroke is not complete in order to prevent the wings from touching the ground. The jump lifts the entire animal high enough to prevent the wings from touching the ground during subsequent wing cycles, when the downstrokes end in the wings held vertically to the longitudinal body axis (Fig. 1R, Y). During two thirds of the downstroke, fore- and hind margins approximately stay at same heights (Fig. 1I,J,P,Q), so the plane of each forewing remains straight to produce maximum uplift. In the last third of the downstroke, the anterior wing margin is put forward and angled compared to the posterior flexible half of the wing, which shows some inertia trailing behind (Fig. 1Q,R,X). To overcome air resistance, the wings during upstroke likewise rotate obliquely along their longitudinal axis when lifted, with the costal margin leading forward and upward, so the wing is held in a vertical position (Fig. 1L, S, AA). Again, the posterior part of the wing slightly cambers and is trailing behind. The anterior area of the wing between veins costa and radius anterior (R1) always remains straight throughout the entire wing cycle, followed by the flexible posterior part of the wing, resulting in a cambering movement throughout the whole wing cycle (see video S1). At no times we could observe any breaking or bending of the wings in the region where the bullae are located.

### Different flight modes in swarming mayflies

In mayflies, the nuptial flight is performed by swarming male specimens, which aggregate in swarms above or near the water body. We video recorded the swarming in *Leptohyphes eximius* (Leptohyphidae) (video S3), *Miroculis fittkaui* (Leptophlebiidae) (video S4), and *Lachlania* sp. (Oligoneuriidae) (video S5)*.* The males of *L. eximius* fly in a vertically orientated pattern, which includes a slower ascent of variable length and a rapid descent in one go. Males of *M. fittkaui* hover more or less stationary, occasionally shooting up very rapidly. In *Lachlania* sp., the males patrol the stream in an irregular horizontal pattern.

### Morphology of wing veins and bullae

**Fig. 2:**
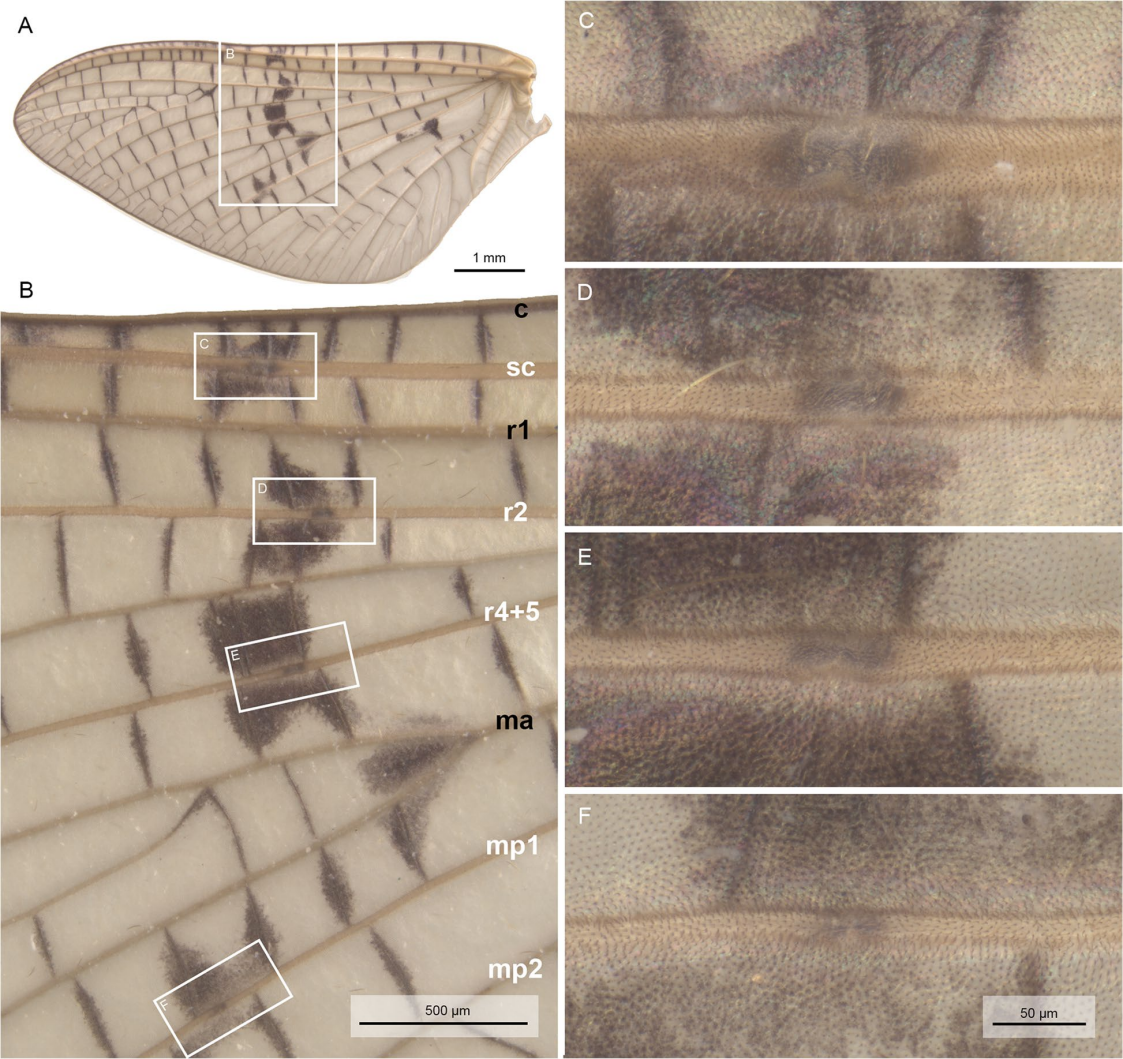
*Ephemera danica* (Ephemeridae), forewing and bullae of subimago in ventral view after critical point drying under light microscopy. **A** Total view, **B** bullae in detailed view, **C** bulla sc, **D** bulla r2, **E** bulla r4 + 5, **F** bulla mp1. Abbreviations of veins (positive veins labeled in black, negative in white): c: costa, sc: subcosta, r: radius, ma: media anterior, mp: media posterior, **C–F** in same magnification

**Fig. 3:**
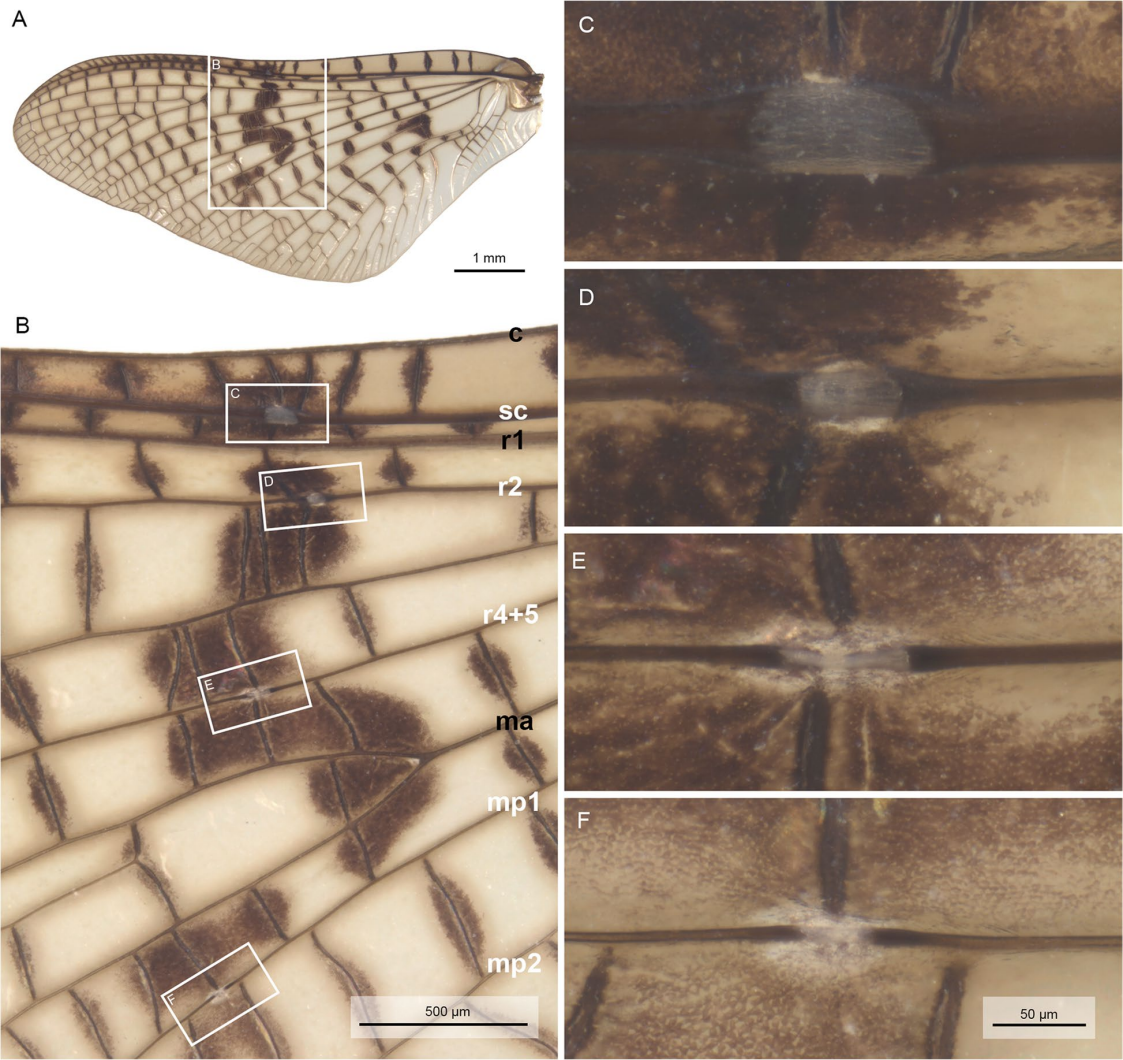
*Ephemera danica* (Ephemeridae), forewing and bullae of imago in ventral view after critical point drying under light microscopy. **A** Total view, **B** bullae in detailed view, **C** bulla sc, **D** bulla r2, **E** bulla r4 + 5, **F** bulla mp1. Abbreviations of veins (positive veins labeled in black, negative in white): c: costa, sc: subcosta, r: radius, ma: media anterior, mp: media posterior. **C–F** in same magnification

Mayfly wings are typically highly corrugated with alternating positive and negative longitudinal veins, which lie either above or below the wing plane, as exemplified here by *Ephemera danica* (Figs. 2A and 3A). When longitudinal veins fork, they retain their spatial position, and an additional intercalary vein appears in between the forked vein to keep the general alternating corrugation. Also, the first three main longitudinal veins (C, Sc, R1) are the most rigid ones, while the following ones tend to get weaker from R2 to the anal veins (Figs. 2B and 3B). Moreover, in some of the negative longitudinal veins (Sc, R2, R4 + 5, MP1) at approximately half-length, there are bullae present as unsclerotized blisters in both subimagines and imagines (Figs. 2 and 3, see also Table 1 for distribution across Ephemeroptera).

**Table 1:** Numbers and distribution of bullae throughout different taxa of extant and fossil mayflies

Family	Species	Sc	R1	R2	R4 + 5	IMA	MP1
**Permoplectoptera**							
Protereismatidae †	*Protereisma insigne*	x	-	x	x	-	x
**Ephemeroptera**							
Siphlonuroidea							
Siphluriscidae	*Siphluriscus chinensis*	x	x	x	x	x	x
Oniscigastridae	*Siphlonella ventilans*	x	x	x	x	-	x
Nesameletidae	*Metamonius anceps*	x	x	x	x	-	x
Rallidentidae	*Rallidens mcfarlanei*	x	x	x	x	-	x
Ameletopsidae	*Chiloporter eatoni*	x	x	x	x	-	x
Dipteromimidae	*Dipteromimus tipuliformis*	x	x	x	x	-	x
Siphlonuridae	*Siphlonurus croaticus*	x	-	x	x	-	x
Acanthametropodidae	*Acanthametropus nikolskyi*	x	-	x	x	-	x
Ameletidae	*Ameletus inopinatus*	x	-	x	x	-	x
	*Metreletus balcanicus*	x	-	x	x	-	x
Metretopodidae	*Siphloplecton *sp.	x	-	x	x	-	x
Ametropodidae	*Ametropus fragilis*	x	-	x	x	-	-
Baetoidea							
Siphlaenigmatidae	*Siphlaenigma janae*	x	-	x	x	-	-
Baetidae: Palaeocloeoninae †	undescribed genus and species	x	-	x	x	-	-
Baetidae: Baetinae	*Baetis rhodani*	x	-	x	x	-	-
Heptagenioidea							
Hexagenitidae †	undescribed genus and species	x	-	x	x	-	-
Coloburiscidae	*Murphyella needhami*	x	-	x	x	-	x
	*Coloburiscus humeralis*	x	-	x	x	-	x
Isonychiidae	*Isonychia berneri*	x	-	x	x	-	x
Oligoneuriidae	*Chromarcys magnifica*	x	-	x	x	-	x
	*Lachlania dominguezi*	-	-	-	-	-	-
	*Oligoneuriella rhenana*	-	-	-	-	-	-
Heptageniidae	*Heptagenia longicauda*	x	-	x	x	-	x
	*Epeorus assimilis*	x	-	x	x	-	x
	*Ecdyonurus venosus*	x	-	x	x	-	x
Leptophlebioidea							
Leptophlebiidae	*Calliarcys humilis*	x	-	x	x	-	x
	*Paraleptophlebia submarginata*	x	-	x	x	-	x
	*Habroleptoides confusa*	x	-	x	x	-	-
	*Thraulodes consortis*	x	-	x	x	-	x
	*Miroculis misionensis* ♀	x	-	x	x	-	x
	*Miroculis misionensis ♂*	x	-	x	x	-	-
Ephemeroidea							
Australiphemeridae †	undescribed genus and species	x	-	x	x	-	x
Potamanthidae	*Potamanthus luteus*	x	-	x	x	-	x
Ephemeridae	*Ephemera danica*	x	-	x	x	-	x
Palingeniidae	*Palingenia longicauda* ♂	x	-	x	x	-	-
	*Palingenia longicauda* ♀	(x)	-	(x)	(x)	-	-
Euthyplociidae	*Euthyplocia hecuba*	x	-	x	x	-	-
Polymitarcyidae	*Ephoron virgo* ♂	x	-	x	x	-	-
	*Ephoron virgo* ♀	-	-	-	-	-	-
	*Campsurus cotaxe*	-	-	-	-	-	-
Behningiidae	*Dolania americana* ♂ / ♀	-	-	-	-	-	-
Ephemerelloidea							
Ephemerellidae	*Eurylophella trilineata*	x	-	x	x	-	x
	*Serratella ignita*	x	-	x	x	-	-
Leptohyphidae	*Leptohyphes cornutus*	x	-	x	x	-	-
Coryphoridae	*Coryphorus aquilus*	x	-	x	x	-	-
Melanemerellidae	*Melanemerella brasiliana*	?	?	?	?	?	?
Teloganellidae	*Teloganella *sp.	?	?	?	?	?	?
Tricorythidae	*Tricorythus discolor*	x	-	x	x	-	-
Machadorythidae	*Machadorythus maculatus*	x	-	x	x	-	-
Dicercomyzidae	*Dicercomyzon marginatum*	x	-	x	x	-	-
Teloganodidae	*Teloganodes *sp.	x	-	x	x	-	-
Ephemerythidae	*Ephemerythus niger*	x	-	x	x	-	-
Vietnamellidae	*Vietnamella thani*	x	-	x	x	-	-
Austremerellidae	*Austremerella picta*	?	?	?	?	?	?
Caenoidea							
Neoephemeridae	*Neoephemera youngi*	x	-	x	x	-	-
Caenidae	*Caenis horaria* ♂	x	-	x	x	-	-
Prosopistomatoidea							
Baetiscidae	*Baetisca rogersi* ♂	x	-	x	x	-	x
Prosopistomatidae	*Prosopistoma variegatum*	-	-	-	-	-	-

[†] denotes extinct taxa, [x] denotes presence of bulla, [-] denotes absence of bulla, [(x)] denotes observable bulla in CLSM only, [?] denotes unclear character state

**Fig. 4:**
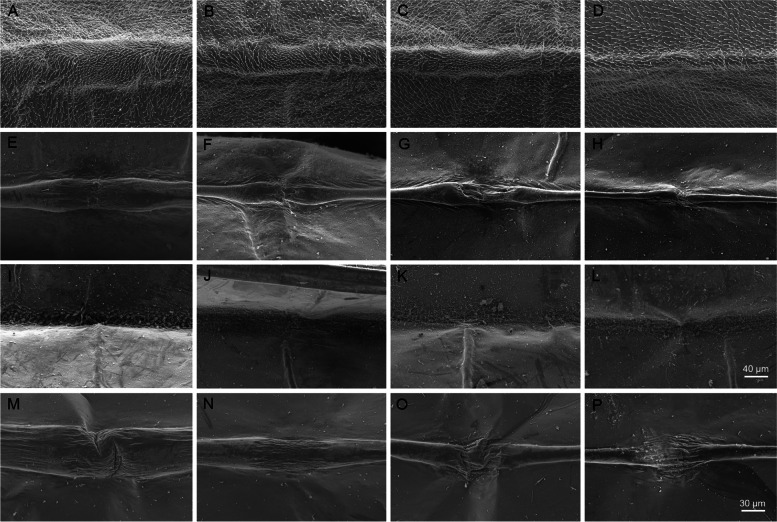
Bullae sc, r2, r4 + 5, and mp1 under SEM, **A–D** *Ephemera danica*, subimago, ventral view, **E–H** *Ephemera danica*, imago, ventral view, **I–L** *Ephemera danica*, imago, dorsal view, **M–P** *Siphlonurus croaticus,* imago, ventral view. **A–L** and **M–P** in same magnification

The membranous nature of the bullae becomes even more evident after critical point drying of the wing (Figs. 2C–F and 3C–F). These desclerotized, membranous portions of the veins appear also inflated under SEM, especially in ventral view (Fig. 4A–H). In dorsal view, bullae in the SEM are much more inconspicuous (Fig. 4I–L). Bullae in principle have a similar structure in different species (e.g. *Siphlonurus croaticus*, Fig. 4M–P).

**Fig. 5:**
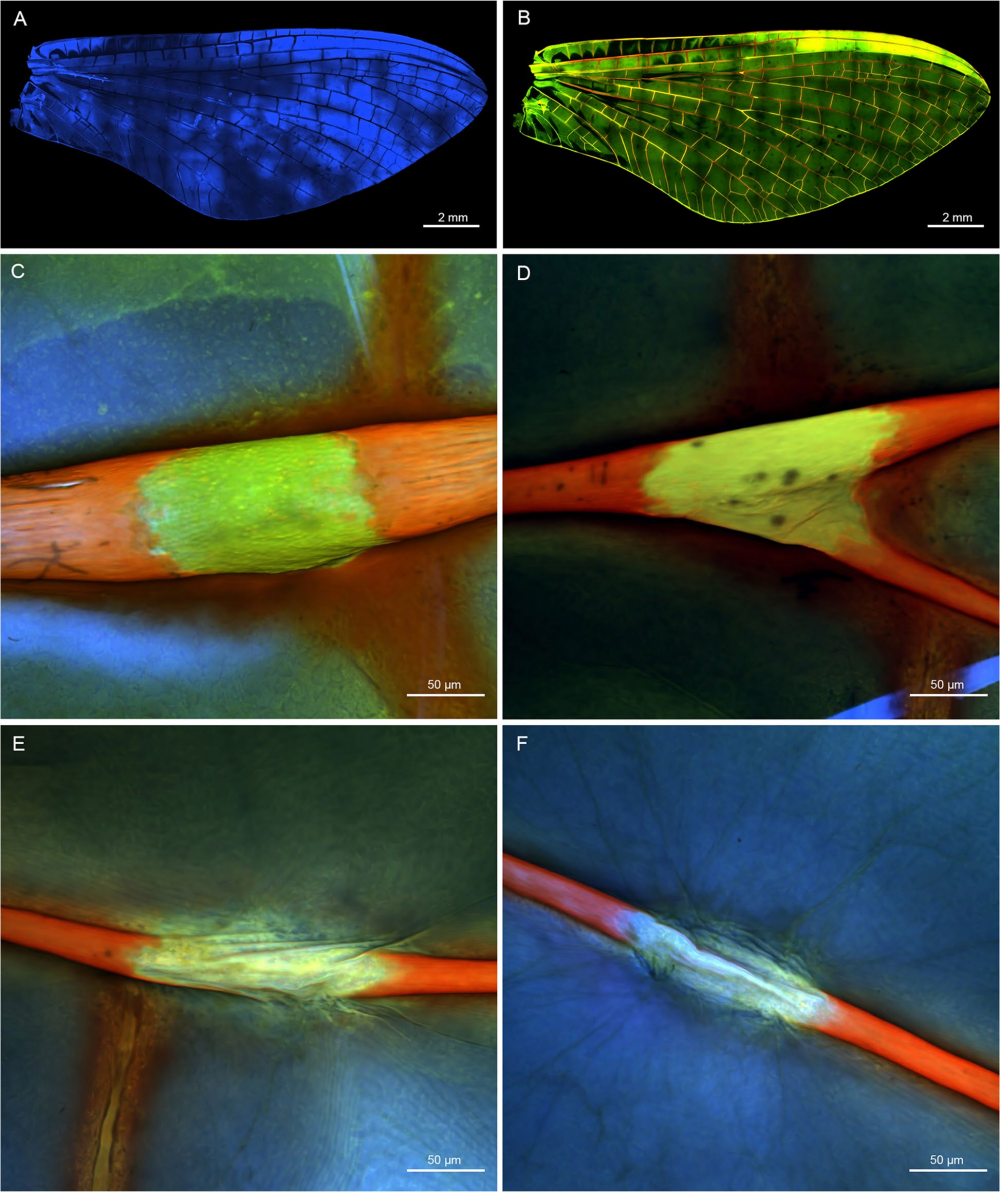
*Siphlonurus lacustris* (Siphlonuridae), imago, ventral view under confocal microscopy. **A** Forewing, presence of resilin, **B** forewing, distribution of sclerotized and membranous areas, **C** bulla sc, **D** bulla r2, **E** bulla r4 + 5, **F** bulla mp1. Red colour indicates sclerotized cuticle, green colour indicates membranous cuticle, blue colour indicates presence of resilin

In confocal laser microscopy, the degree of resilin enrichment in the cuticle of *Siphlonurus lacustris* can be visualized (Fig. 5A) by the intensity of blue colour. Also in all other investigated species, resilin is distributed all over the wing membrane, but neither particularly enriched in the main longitudinal veins nor in the bullae themselves. Membranous and sclerotized properties of the wing veins can also be visualized by different colours, where membranous areas are shown in green colour and sclerotized areas in red colour. It becomes obvious that the sclerotized exocuticular layer is entirely missing in the bullae ventrally (Fig. 5B–F).

**Fig. 6:**
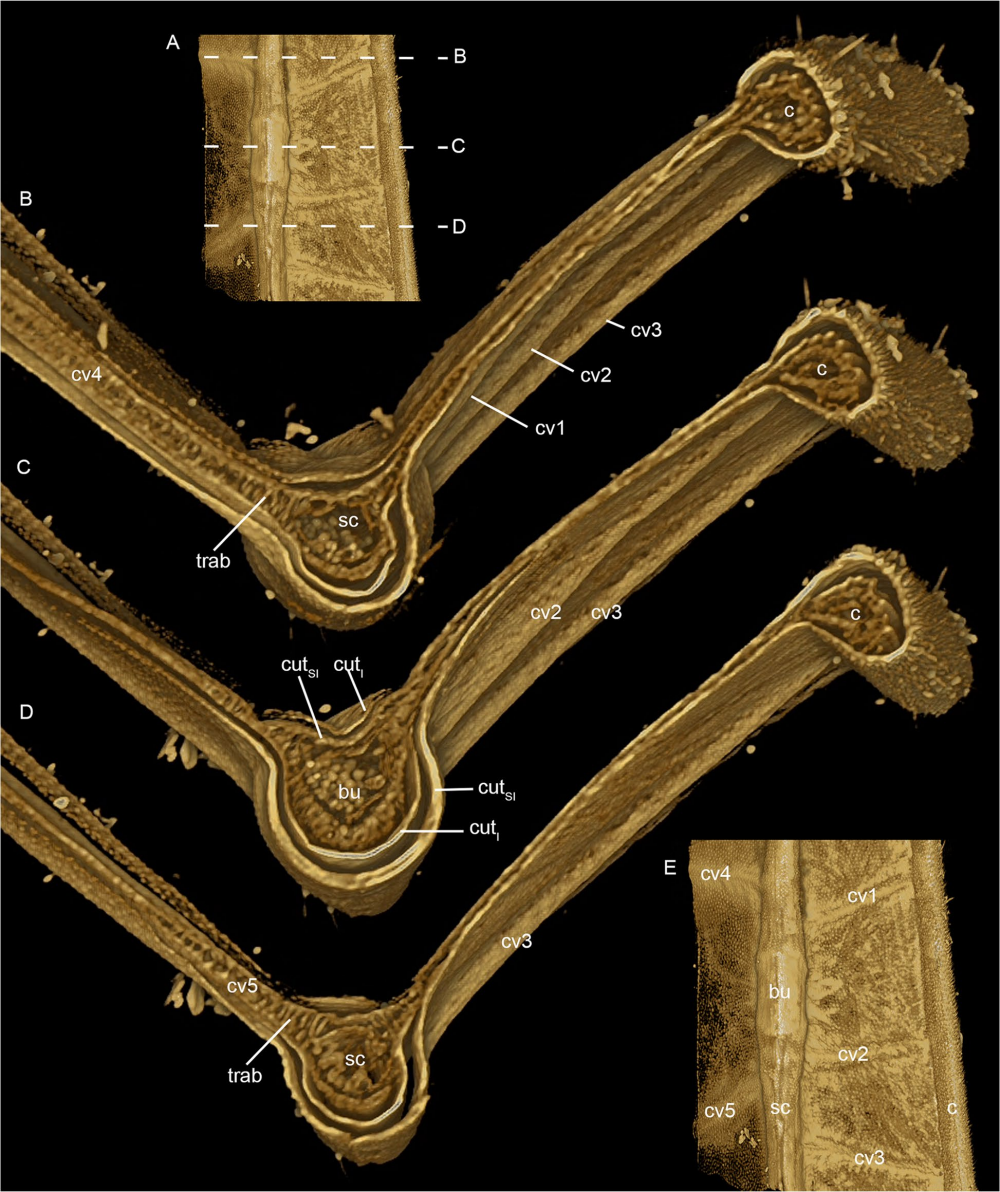
*Ephemera danica* (Ephemeridae), subimago, region of bulla sc shown as volume rendering based on µCT data. **A** ventral view, dashed lines indicate different levels of cross cuts in **B–D**, **E** ventral view with longitudinal veins, crossveins and bulla sc indicated. Abbreviations: c: costal vein, sc: subcostal vein, cv1-3: crossveins in costal field, cv4-5: crossveins in subcostal field, bu: bulla, cut_SI_: subimaginal layer of cuticle, cut_I_: imaginal layer of cuticle, trab: trabeculae. Without scales

µCT scans of the subcostal vein in *E. danica* reveal that its ventral layer is much thicker than its dorsal counterpart in both subimago (Fig. 6B–D) and imago. The same applies to all other negative longitudinal veins. In contrast, in positive veins it is always the dorsal layer, which is thickened. The only exception is the costal vein, which is in the leading edge of the wing and therefore equally thickened throughout (Fig. 6B–D).

Additionally, in the subimago, the imaginal wing is already preformed and visible within the surrounding subimaginal wing, showing already the same pattern of sclerotization of cuticle. At this developmental stage, between the upper and lower cuticles of crossveins there are multiple dorsoventral, column- or strut-like connections present, reminiscent of palisade parenchyma in plant tissue or trabeculae in vertebrate bones (Fig. 6B, D). These trabeculae are missing after the final moulting in the aerodynamic profile of the thinner imaginal wing. The resolution of the µCT is not sufficient to determine the nature of these trabeculae. As a more detailed investigation on the histology of the wing was beyond the scope of this contribution, we will delve into this matter in a forthcoming separate study.

### Distribution of bullae in different extant families of mayflies

**Fig. 7:**
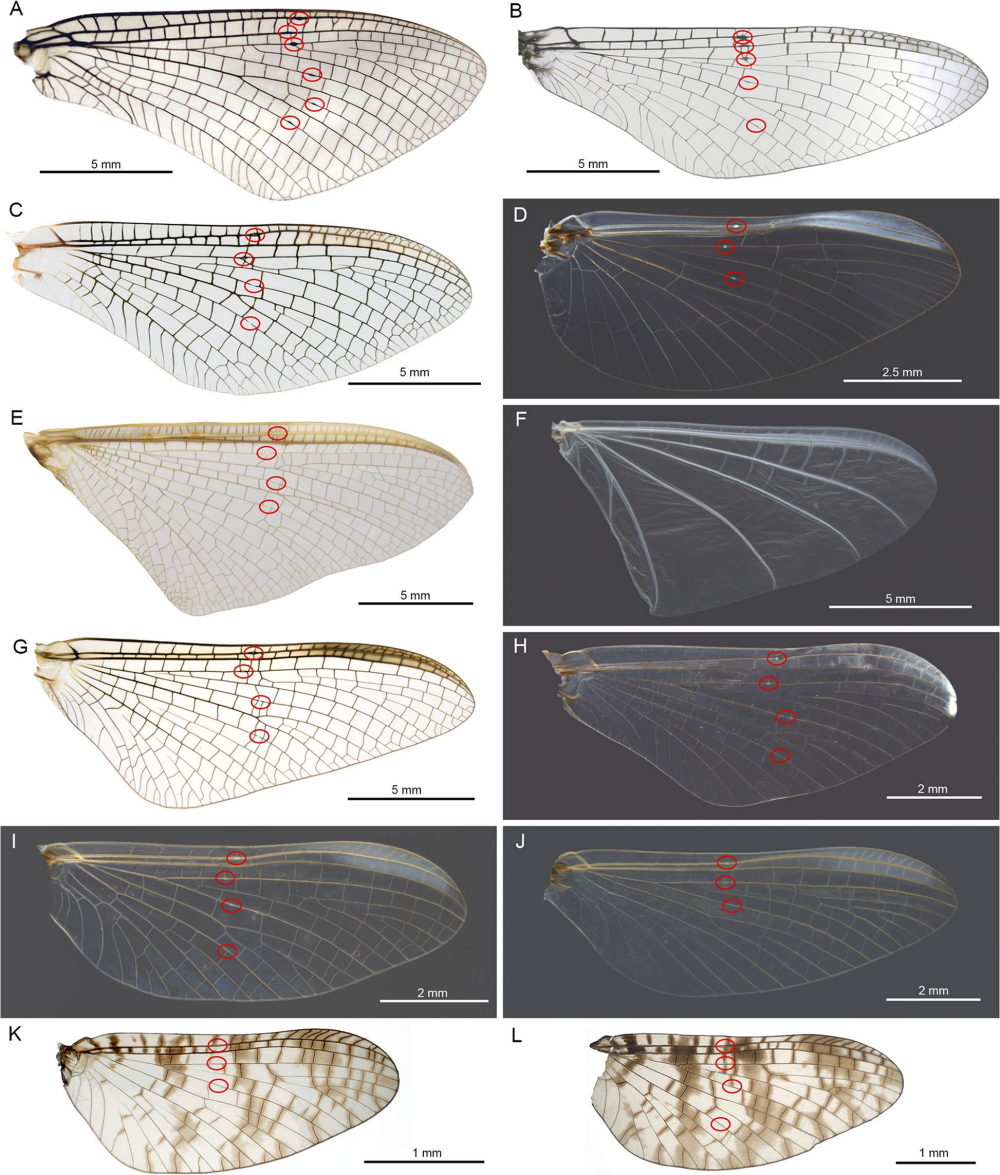
Distribution of bullae in forewings of species from different families. **A** *Siphluriscus chinensis* (Siphluriscidae), **B** *Metamonius anceps* (Nesameletidae), **C** *Siphlonurus croaticus* (Siphlonuridae), **D** *Baetis rhodani* (Baetidae), **E** *Chromarcys magnifica* (Oligoneuriidae: Chromarcyinae), **F** *Oligoneuriella rhenana* (Oligoneuriidae: Oligoneuriinae), **G** *Ecdyonurus venosus* (Heptageniidae), **H** *Isonychia berneri* (Isonychiidae), **I** *Calliarcys humilis* (Leptophlebiidae: Calliarcyinae), **J** *Habroleptoides confusa* (Leptophlebiidae: Leptophlebiinae), **K** *Miroculis misionensis* ♂ (Leptophlebiidae: Atalophlebiinae), **L** *Miroculis misionensis* ♀ (Leptophlebiidae: Atalophlebiinae)

**Fig. 8:**
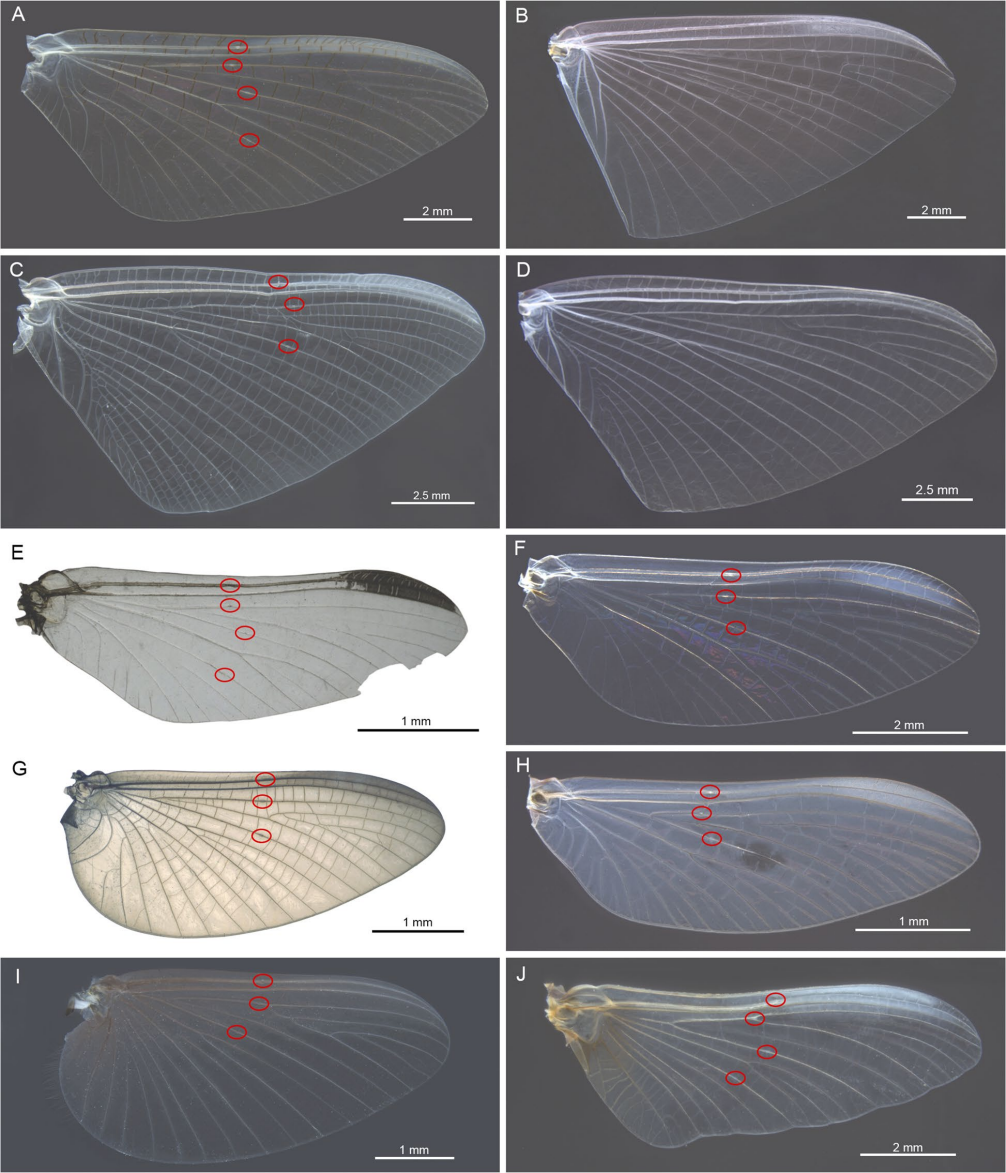
Distribution of bullae in forewings of species from different families. **A** *Potamanthus luteus* (Potamanthidae), **B** *Dolania americana* ♂ (Behningiidae), **C** *Ephoron virgo* ♂ (Polymitarcyidae), **D** *Ephoron virgo* ♀ (Polymitarcyidae), **E** *Eurylophella trilineata* (Ephemerellidae), **F** *Serratella ignita* (Ephemerellidae), **G** *Leptohyphes cornutus* (Leptohyphidae), **H** *Teloganodes* sp. (Teloganodidae), **I** *Caenis horaria* (Caenidae), **J** *Baetisca berneri* (Baetiscidae)

We studied the distribution of bullae in numerous species of mayflies throughout the order, representing the presently recognized families within Ephemeroptera (Figs. 7 and 8, Table 1).

Within Siphlonuroidea, in Siphluriscidae (Fig. 7A) there are six bullae present, approximately at half-length in each of the following veins Sc, R1, R2, R4 + 5, iMA, MP1 (in order from anterior to posterior). All of these veins except R1 are negative veins. *Siphluriscus chinensis* is the only mayfly, in which we found a clear bulla in iMA. In siphlonuroid families with amphinotic distribution, e.g. in Nesameletidae (Fig. 7B), Oniscigastridae, and Ameletopsidae, bullae are present in the same veins except of iMA. This also applies for Rallidentidae and Dipteromimidae, which are endemic to New Zealand and Japan, respectively. In other siphlonuroid families, which are distributed in the northern hemisphere, there are neither in iMA nor in R1 bullae present, e.g. in Siphlonuridae (Fig. 7C), Ameletidae, Metretopodidae, and Acanthametropodidae. In Ametropodidae, there are only three bullae present, namely in Sc, R2, and R4 + 5.

In Baetoidea, in both Siphlaenigmatidae and Baetidae (Fig. 7D), there are bullae present in Sc, R2, and R4 + 5.

In Heptagenioidea, we found four bullae in Coloburiscidae, Isonychiidae (Fig. 7H), Oligoneuriidae: Chromarcyinae (Fig. 7E), and Heptageniidae (Fig. 7G). In the highly modified wings of Oligoneuriidae: Oligoneuriinae (Fig. 7F), there were no bullae found at all.

In Leptophlebioidea, four to three bullae were found in different species in each of the different subfamilies Leptophlebiinae and Atalophlebiinae: While *Calliarcys humilis* (Fig. 7I) and *Paraleptophlebia submarginata* (both Leptophlebiinae) both have four bullae, there are only three bullae present in *Habroleptoides confusa* (Fig. 7J). Likewise, in *Thraulodes consortis* (Atalophlebiinae), there are four bullae present, while both sexes of *Ulmeritus carbonelli* have only three bullae. There may be also sexual dimorphism present like in *Miroculis misionensis*, in which females (Fig. 7L) are equipped with four, males (Fig. 7K) only with three bullae.

In Ephemeroidea, four bullae were found in Potamanthidae (Fig. 8A) and Ephemeridae (Fig. 3A), while in Euthyplociidae only three bullae are present. In Palingeniidae, males have three bullae, while the three bullae in females are more subtle and largely reduced. Sexual dimorphism can also be found in Polymitarcyidae, where males of *Ephoron virgo* have three bullae (Fig. 8C), while in females no bullae are obvious (Fig. 8D). In other species like *Campsurus cotaxe*, bullae are absent in both sexes. No bullae were also found in Behningiidae (Fig. 8B).

In Ephemerelloidea, most of the families have only three bullae, like in Leptohyphidae (Fig. 8G), Coryphoridae, Teloganellidae, Teloganodidae (Fig. 8H), Tricorythidae, Machadorythidae, Dicercomyzidae, Ephemerythidae, and Vietnamellidae. In Ephemerellidae, except of the usual three bullae (e.g. *Serratella ignita*, Fig. 8F) there were four bullae found in *Eurylophella trilineata* (Fig. 8E). Due to the poor conservation of the only known adult specimen of *Melanemerella brasiliana*, we were not able to verify the number of bullae in Melanemerellidae. Likewise, we had no access to *Austremerella picta* nor to any species of *Teloganella* sp., so we have no information on the bullae in Austremerellidae and Teloganellidae.

In Caenoidea, in both Neoephemeridae and Caenidae (Fig. 8I), there are three bullae present, in some cases only very subtle or even not recognizable.

In Prosopistomatoidea, Baetiscidae (Fig. 8J) have four bullae, while in the highly modified wings of Prosopistomatidae bullae are absent in both sexes.

### Presence of bullae in fossil Ephemeroptera and Ephemerida

**Fig. 9:**
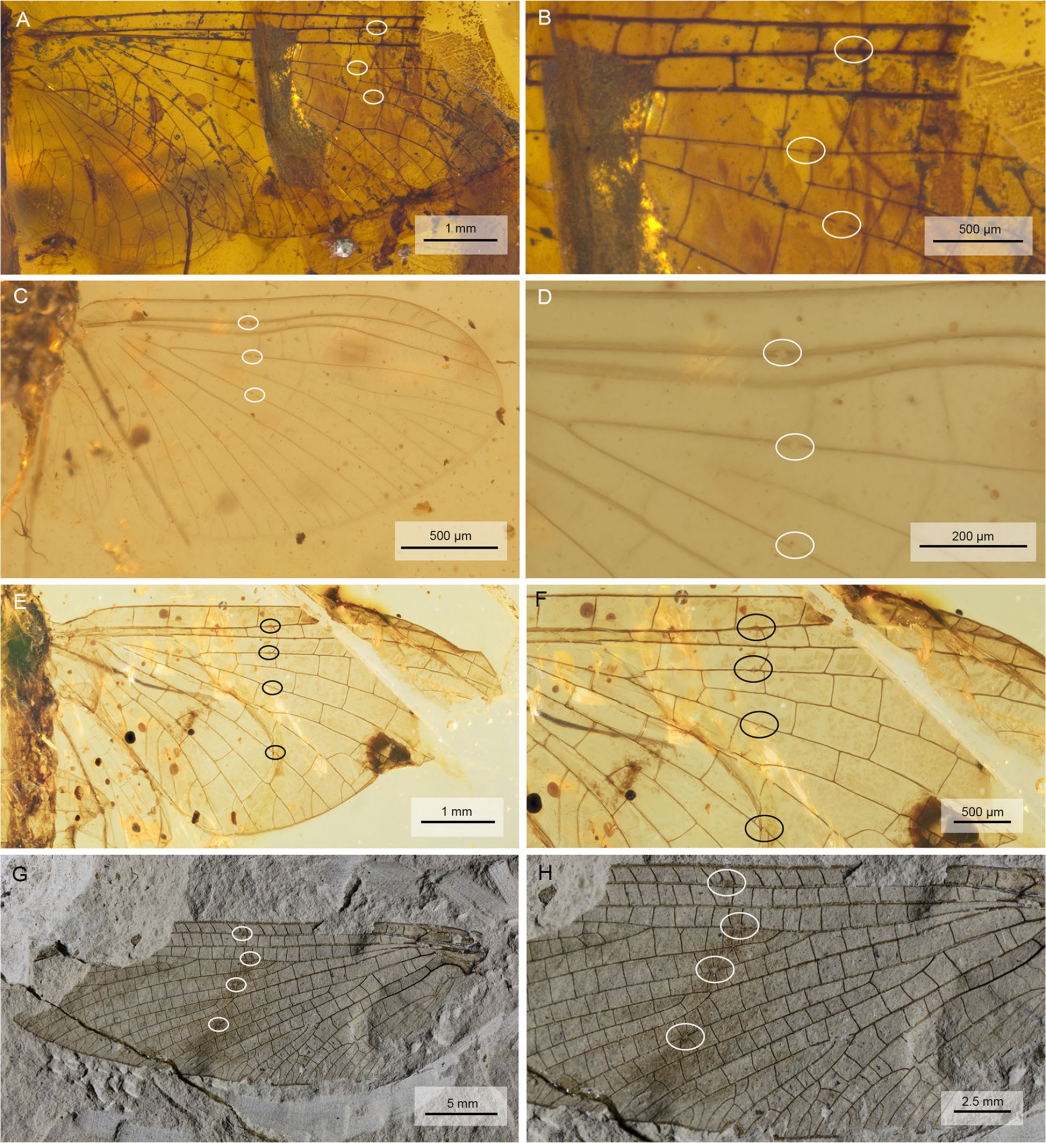
Bullae in wings of fossil crowngroup and stemgroup mayflies. **A, B** Undescribed species of Hexagenitidae † (Ephemeroptera), forewing. Burmese amber, Mid-Cretaceous, ca. 99 ma. **C, D** Undescribed species of Baetidae: Palaeocloeoninae † (Ephemeroptera), forewing. Burmese amber, Mid-Cretaceous, ca. 99 Ma. **E, F** Undescribed species of Australiphemeridae † (Ephemeroptera), forewing, first record from Lebanese amber, Lower Cretaceous, ca. 129 Ma. **G, H** holotype of *Protereisma insigne* † (Ephemerida: Permoplectoptera: Protereismatidae), hind wing, Lower Permian of Kansas, ca. 272 Ma., courtesy of the Yale Peabody Museum, Division of Vertebrate Paleontology, Yale University, Peabody.yale.edu. Photographs by S. Butts, 2023

We checked the wings of different fossil species of extant families for the presence of bullae, e.g. among others *Borinquena parva* (Leptophlebiidae) in Dominican amber, *Siphloplecton* sp. (Metretopodidae) in Baltic Amber, and *Burmella paucivenosa* (Vietnamellidae) in Burmese amber. All of them displayed just the same number and distribution of bullae like their extant relatives. We also documented the presence of bullae in extinct families and subfamilies of Ephemeroptera. Undescribed species of Hexagenitidae and Baetidae: Palaeocloeoninae in Burmese amber (99 Ma) were equipped with bullae in Sc, R2, and R4 + 5 (Fig. 9A–B and C–D), respectively. We here also record for the first time an undescribed species of Australiphemeridae in Lebanese amber (129 Ma) that bears bullae in Sc, R2, R4 + 5, and MP1. Finally, we were also able to verify the presence of bullae in stemgroup Ephemerida. The hind wing of *Protereisma insigne* (Permoplectoptera: Protereismatidae) from the Lower Permian of Kansas (272 Ma) shows likewise clearly pronounced bullae in Sc, R2, R4 + 5, and MP1 (for details, see Fig. 9G,H, Table 1).

### Subimaginal moulting and wing extraction

**Fig. 10:**
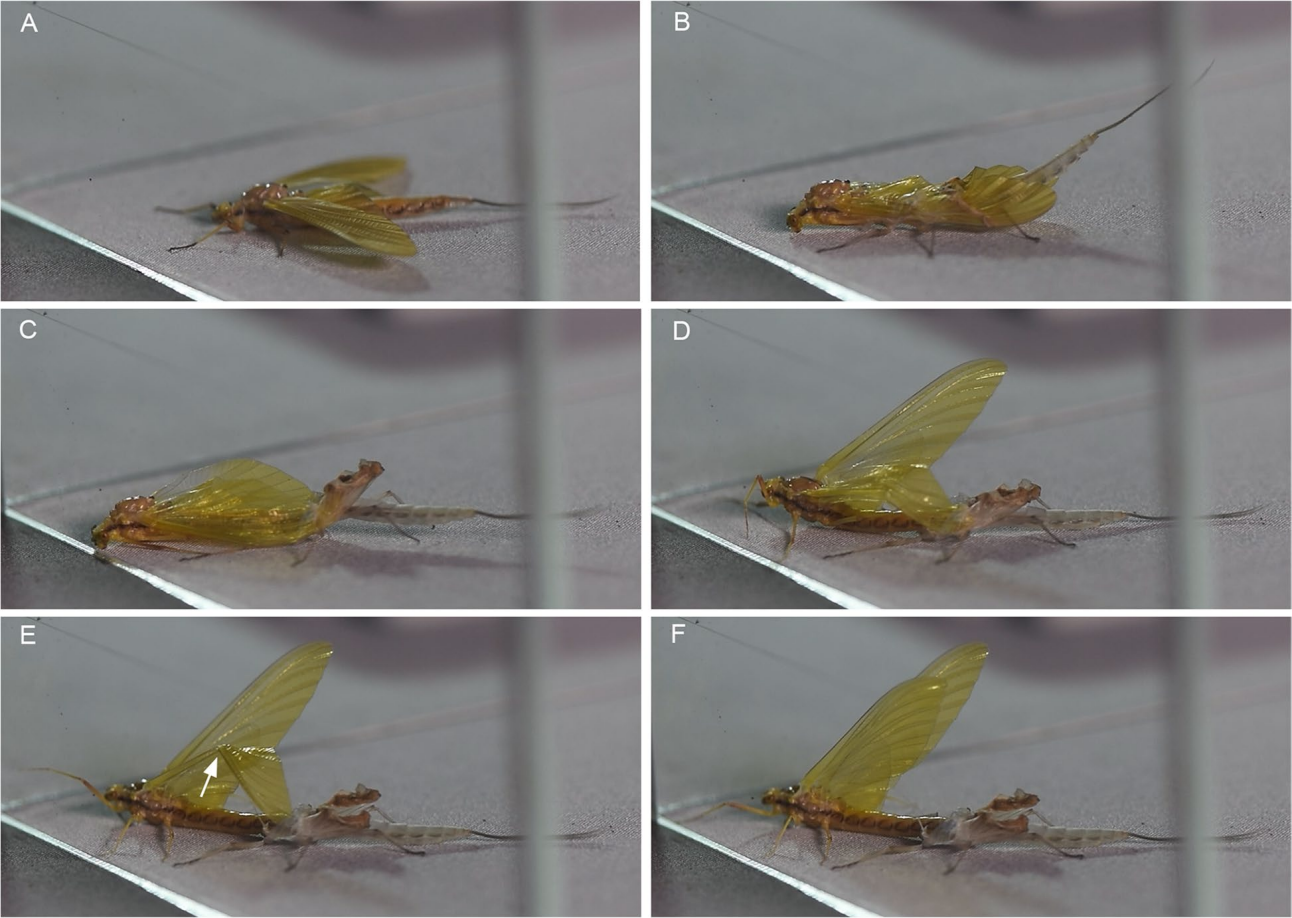
**A–F** Different moulting stages from subimago to imago in *Chiloporter eatoni* (Ameletopsidae), still photographs from video S8. Arrow in **E** shows wing bending at line predetermined by bullae

**Fig. 11:**
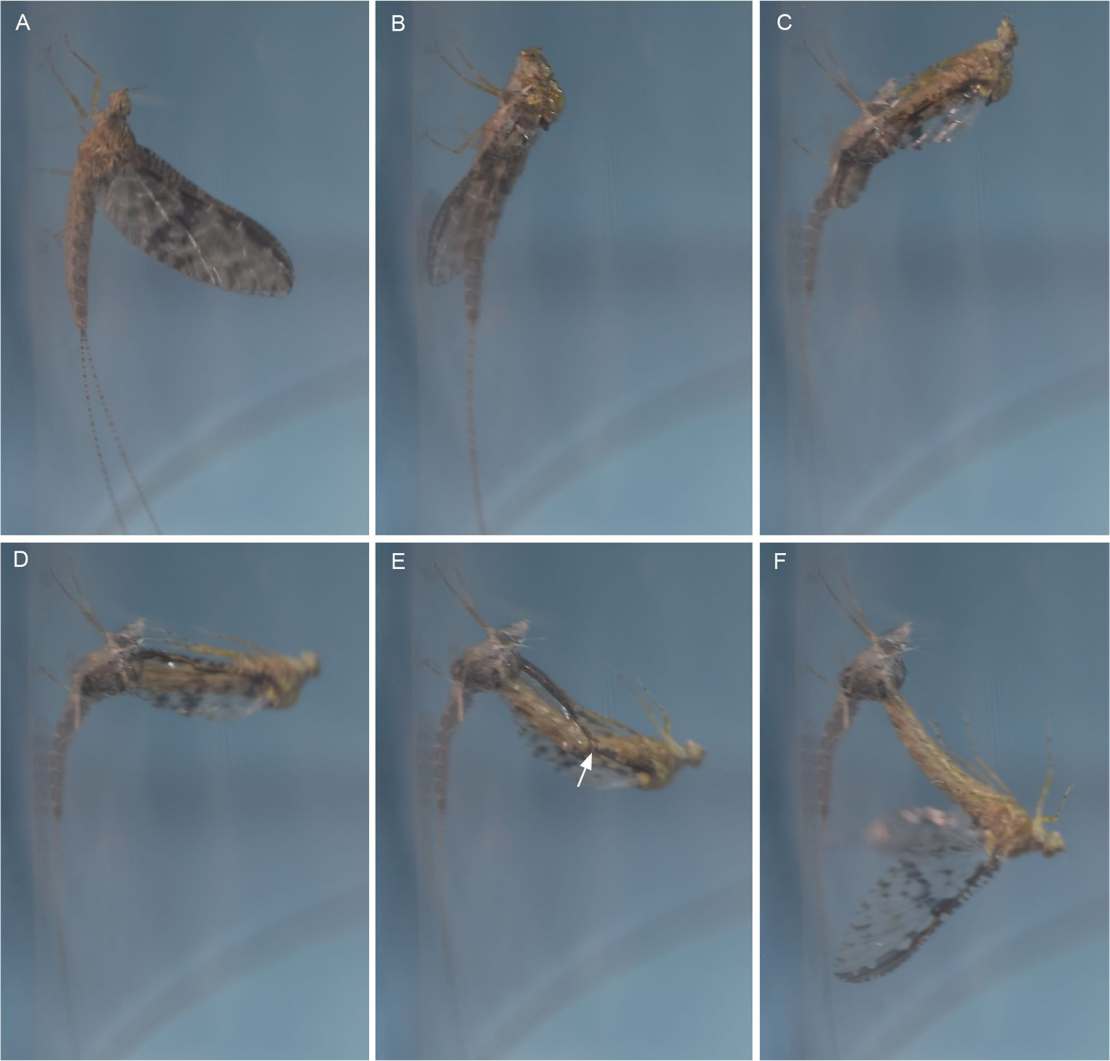
**A–F** Different moulting stages from subimago to imago in *Callibaetis guttatus* (Baetidae), still photographs from video S9. Arrow in **E** shows wing bending at line predetermined by bullae

**Fig. 12:**
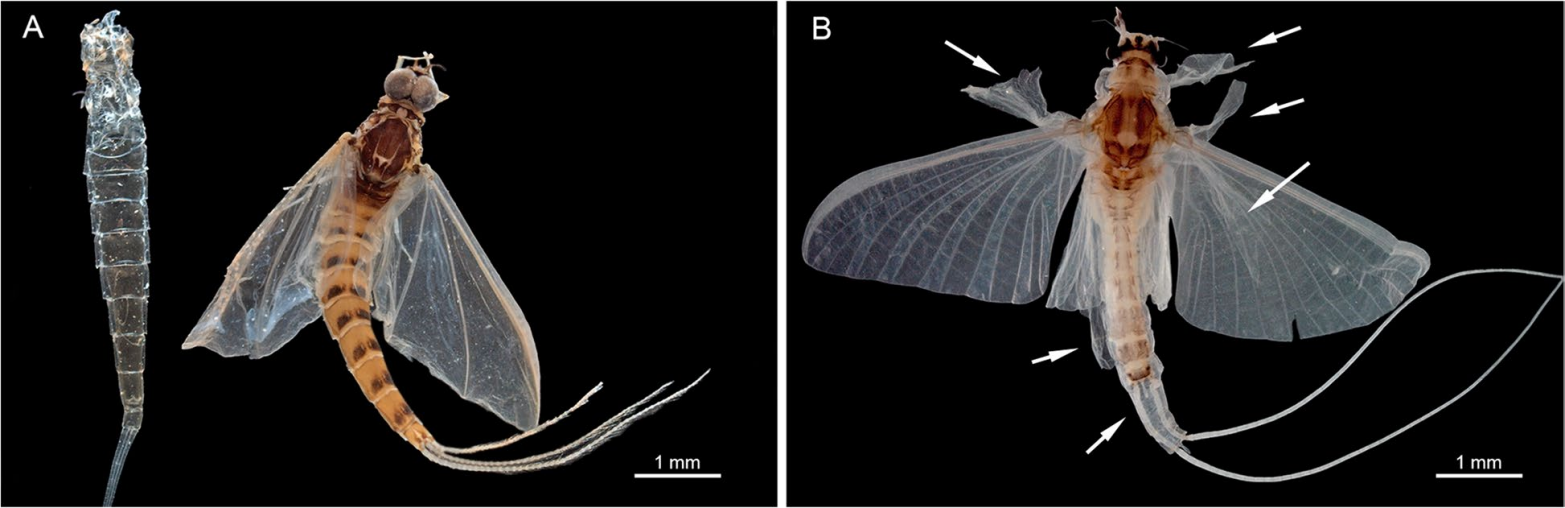
Modified moulting in species lacking bullae. **A** Body moult in *Homoeoneuria* sp. (Oligoneuriidae) with subimaginal wings remaining unshed. **B** Additional delamination and fragmentation of subimaginal wing cuticle in *Tortopsis sarae* (Polymitarcyidae). Arrows point to abdominal exuvia and delaminating wing cuticle

To observe the process of wing moulting and the possible role of bullae therein, subimaginal moultings were video recorded in *Chiloporter eatoni* (Ameletopsidae) (Fig. 10, video S8), *Siphlonurus croaticus* and *Siphlonurus lacustris* (Siphlonuridae), *Callibaetis guttatus* (Fig. 11, video S9) and *Baetodes huaico* (Baetidae), *Hapsiphlebia anastomosis, Nousia bella* and *Thraulodes consortis* (Leptophlebiidae), *Ephemera danica* (Ephemeridae) (video S10), *Lumahyphes guacra* (Leptohyphidae) and *Caenis gonseri* (Caenidae). Additionally, we observed some aberrant modes of subimaginal moulting in *Oligoneuriella rhenana* and *Homoeoneuria* sp. (Oligoneuriidae) (Fig. 12A), and *Asthenopus curtus* and *Tortopsis sarae* (Polymitarcyidae) (Fig. 12B), or even loss of subimaginal moulting in the females of *Ephoron virgo* (Fig. 8D) and *Palingenia longicauda* (Palingeniidae).

In *Callibaetis guttatus*, the subimago usually emerges from the last nymphal instar in the afternoon between 3.30 and 5 pm local time (ART, GMT-3). It immediately flies to a nearby place and rests until the subimaginal moult, which takes place on the same night; usually well before dawn between 3 and 4am. The subimago clings to the vegetation in a vertically orientated position with the head up and becomes inactive for some time. Immediately before moulting the animal gets restless performing sudden movements. The actual moulting process (Fig. 11) is initiated by an irregular trembling of the entire animal and by flickering of the wings, which eventually change their posture (see Video S9). From initially being held vertically above the abdomen, the wings are gradually taken down horizontally and then even further backwards and downwards, so that the costal vein is horizontally in line with the abdomen and the wing tip directed posteriorly. In this way, the wings are aligned to the longitudinal body axis to enable an unobstructed, smooth extraction of the imaginal wing. Additionally, the legs are taken backwards to become likewise aligned. At the same time, the thoracic cuticle ruptures dorsomedially along with the epicranial suture of the head, as the emerging imago propels itself forward by peristaltic body movements. As the animal has almost shed its entire body except for the wing tips, tarsi, last abdominal segments, and tail filaments, it bends over its back with the head down. To facilitate the moulting of the wing apex, it then starts lifting its wings. Thereby the entire wings bend along a flexion line predetermined by the position of the bullae. As soon as the wing tip is released, the wing immediately snaps back in its original shape. At the same time, while the subimaginal leg cuticle remains in natural position with the subimaginal claws anchored to the substrate, the imaginal legs are pulled out. Thereby the imaginal legs change from their natural position in a way that all segments of each leg are aligned in a straight line and directed backwards. As soon as the imaginal legs are fully pulled out, they immediately return to their natural position with usual angles between the different leg segments of each leg to become fully functional again. With the imaginal legs in function, the imago bends upwards again and returns to its initial upright position. As a final step, the tail filaments are extracted from the remaining subimaginal cuticle. The same mode of moulting was basically also observed in all other species mentioned above (see also video S10 for *Ephemera danica*).

In some cases, like in *Chiloporter eatoni*, under the artificial conditions provided, the animal did not have the choice to moult in a vertical position, but was forced to moult in a horizontal position (Fig. 10, Video S8). Still, the entire moulting process was similar to the one observed in *C. guttatus* except that the animal did not bend backwards. During moulting, the animal likewise propelled forward, thereby pulling out imaginal wings and legs of the subimaginal cuticle. However, the extraction of the imaginal wing took much more time and appeared to require more effort than moulting in the upright position.

In any case, the bullae determine the position where the wings break in the critical moment of the extraction of the imaginal wing from within the subimaginal one. Nevertheless, the imaginal wing still breaks in the same region even when there are no evident bullae present, like in *C. gonseri*.

Apart from the usual process of moulting, different modes exist in some taxa: In *Oligoneuriella rhenana* and *Homoeoneuria* sp. (Oligoneuriidae), only the body sheds its subimaginal cuticle, while the subimaginal wings remain unshed like an envelope around the imaginal ones (Fig. 12A). In *Asthenopus curtus* and *Tortopsis sarae* (Polymitarcyidae) (Fig. 12B), additionally to the body moulting, the subimaginal wing cuticle is delaminated, separates and peels off during flight. In some other species like *Dolania americana* (Fig. 8B), *Ephoron virgo* (Fig. 8D), or *Palingenia longicauda*, the females do not moult at all and remain in the subimaginal stage throughout their winged life.

### Cardboard paper models

To test the behaviour of the wing, the wing cardboard model was fixed at its base by holding it firmly with one hand. When mechanical pressure was applied to the apical half from dorsally with the other hand, it bent following a line predetermined by the positions of the bullae. Applying approximately the same amount of mechanical pressure from ventrally, the model was not affected, withstood the pressure and would not bend at all. Applying increased pressure at some point resulted in random bending of the wing (video S11).

## Discussion

### There is no correlation between different types of flight and numbers of bullae

Several authors [20, 30, 31] had suggested a relationship between the presence of bullae and a vertically orientated mating flight. After studying the mating flight of several species of *Miroculis* (Leptophlebiidae), Domínguez & Abdala [33] found that males of different species performed different types of flights such as static hovering or fast upward shooting flight, despite having identical sets of bullae. In *Miroculis fittkaui*, the males even perform both types of flight (see Video S4). This suggests that there is no correlation between up and down flight and the absence or different numbers of bullae. Furthermore, although female mayflies generally do not participate in the vertical swarming and only rarely fly a pronounced up and down pattern, e.g. for oviposition, they still have bullae. On the contrary, there are species with bullae (*Leptohyphes eximius*, Leptohyphidae) and others without evident bullae (*Caenis ludicra*, Caenidae), which still both perform an apparently similar up and down flight pattern (see also Video S3), leaving the bullae rather irrelevant for a specific type of flight. Likewise, subimagines do not participate in vertically directed mating flights, but still do have bullae. So, from all data available nothing points to a correlation between different types of flight and arrangements of bullae.

### There is no wing bending mediated by bullae during flight

The influential hypothesis of Edmunds & Traver [20] of wing bending along a flexion line determined by bullae during upstroke was also supported by Brodsky [30], p. 94, Fig. 5.9a]. He indicated two transversal flexion lines through the bullae that together would allow wing tip deflection in the same way that Edmunds & Traver [20] had proposed. This was adopted by Wootton [31], who used simplified paper models to comprehend the mechanics of an alleged wing bending during flight. His paper models indeed performed exactly in the way as predicted by Edmunds & Traver [20]. Our own paper models, in which we meticulously tried to reproduce the actual wing corrugation, likewise performed as predicted (Video S11). Nevertheless, our own observations on the wing posture during wing cycles indeed confirm that there is no bending of the apical half of forewing during upstroke. This directly implies that bullae do not facilitate wing bending during flight, thus leaving their actual function in limbo. Domínguez & Abdala [33] hypothesized that the presence of these unique structures might somehow be linked to the likewise unique subimaginal stage in mayflies.

### Bullae are desclerotized wing areas defining a predetermined bending line during moulting

Wing corrugation in mayflies is a structural necessity for stable flight. Additional stability is provided by the sclerotization of the main longitudinal wing veins. In most mayflies, the first three longitudinal veins (C, Sc, R1) are significantly more rigid than the remaining ones (Figs. 2 and 3). Our high-speed videos (Videos S1, S2) show that the wings move in vertical direction during upstroke, in this way minimizing air resistance, the brunt of which is taken by the leading wing edge before moving anteriorly and later down in the downstroke. In this way, the three veins work together as a functional unit during upstroke, providing rigidity and stability for the entire wing despite the presence of bullae as potential weakening points. The latter are however irrelevant during the vertical upstroke, when the longitudinal veins are vertically aligned and the air resistance is overcome by a rigid costal vein in the lead, supported by the likewise rigid subcostal and first radial vein.

According to our observations, bullae do not play a significant role in flight. Rather it seems that they are a prerequisite for wing moulting, aiding the extraction of the already rigid and functional imaginal wing from the subimaginal cuticle.

When the subimago sheds its wings, halfway through the process it lifts its wing, thereby trying to get rid of the remaining subimaginal cuticle and to fully extract the imaginal wing. With this movement, the wing tip experiences a ventrally directed force, which puts tensile load on the positive veins and pressure load on the negative veins. In order to facilitate a ventrally directed bending of the wing during moulting, bullae in the main negative veins would be able to give in to the pressure load at a predetermined point of least resistance without damaging the veins during bending at wing extraction. In this way, the bullae altogether predetermine the flexion line necessary for the undamaged extraction of the imaginal wing from the subimaginal cuticle.

The asymmetrical sclerotization of the longitudinal veins (see Fig. 6) halves their weight, while not compromising their structural integrity. The sclerotization always remains on the outer, most exposed levels of the wings (that is the dorsal side in positive veins, but ventral ones in the negative veins), which are more likely to encounter external forces, thereby contributing to a mechanical protection of the delicate wing membrane. Our observations contradict Horstmann’s [34] findings, as we did not observe any particular enrichment of resilin in bullae, along flexion lines, or in veins. Instead, resilin is distributed throughout the entire wing membrane, most likely contributing to its elasticity and resilience.

Just after the last-instar nymph has moulted to the subimago, palisade- or parenchyma-like trabeculae were observed in the imaginal wing within the subimaginal cuticle, which seem to obliterate before the imaginal moulting. Due to the limited resolution of the µCT, we were not able to determine the nature of these trabeculae, but we assume these are hypertrophied epidermal cells. It remains to be clarified if these trabeculae are temporal structures providing rigidity to enable the instant flight of the just moulted, still soft-winged subimago.

### Evolutionary tendencies of bullae within Ephemeroptera

The presence of bullae is generally correlated to the moulting of functional wings. Mayflies are the only extant pterygote insect order in which a winged subimaginal stage moults its wings: so far, no bullae have been observed in any other pterygote insect order.

However, there is a general tendency within mayflies to diminish the number of bullae. Phylogenetically, *Siphluriscus chinensis* is generally regarded as sister-group to all other extant mayflies [35]. It is the only mayfly with six bullae, which is the highest number of bullae throughout the order. In the remaining “Siphlonuroidea,” a probably paraphyletic assemblage of basal families, it can be seen that the amphinotic families (Oniscigastridae, Nesameletidae, Ameletopsidae), the endemic New Zealand family Rallidentidae, and the Japanese family Dipteromimidae have lost bulla IMA. Further reductions occur in siphlonuroid families of the Northern Hemisphere (Siphlonuridae, Ameletidae, Metretopodidae, Acanthametropodidae), finally leading to only three bullae in Ametropodidae and also in Baetoidea (see Table 1), which according to Ogden et al. [35] represent the second basal split within mayflies.

Also, in some mayfly groups the females show a reduction or loss of bullae, which is highly correlated with their loss of imaginal moulting. Within Ephemeroidea, even though the bullae in females of *Palingenia longicauda* are still visible, they appear far more subtle than in their male counterparts, which still undergo moulting. The males of *Ephoron virgo* do moult as well and have three bullae, while in females, which remain in the subimaginal stage, no bulla can be observed (see Table 1).

Some other mayflies have developed different modes of imaginal moulting: In some Polymitarcyidae, e.g. *Asthenopus curtus* and *Tortopsis sarae*, the subimaginal cuticle is delaminated during moulting, peeling off like sun-burned skin. In this case, the modified moulting is correlated to the total loss of bullae in both sexes. Likewise, in many species of Oligoneuriidae (e.g. *Oligoneuria rhenana*, *Lachlania dominguezi, Homoeoneura* sp.), only the body is shed and the subimaginal cuticle remains on top of the imaginal wing. In all these species, this is always correlated with the total loss of bullae.

There are also some taxa like *Prosopistoma* sp., the males of which do moult from subimago to imago [36], but still their wings have no visible bullae. However, this correlates with less pronounced wing corrugation, accompanied by a loss of crossveins. It may be well possible that in this genus the diminished wing corrugation enables a full extraction of the imaginal wing even without the presence of bullae. Finally, within the same genus of some Leptohyphidae and Caenidae, there may be larger species with stronger bullae (e.g. *Leptohyphes cornutus, Caenis horaria*) and smaller species with weaker ones (e.g. *Leptohyphes eximius, Caenis gonseri*), so there also seems to be a correlation between wing size and development of bullae.

However, with the highly specialized mayfly *Dolania americana* (Behningiidae) we have encountered one exception, which does not fit into our conclusions. Both male and female wings do not have bullae. While the females remain in the subimaginal stage as expected, the males surprisingly do moult without the presence of wing bullae [37]. This is unexpected, because it is not a small species, and neither do the wings lack corrugation nor they are lacking crossveins. It remains to be investigated if the moulting of *Dolania americana* follows the same pattern like described in most other mayflies or if they have developed a different type of imaginal moulting without necessity for wing bending.

### Evolutionary significance of bullae in pterygote insects

Unlike pterygote insects, primarily apterygote hexapods like Entognatha, Archaeognatha, and Zygentoma continue moulting even as sexually mature adults [2, 9]. Only with the development of wings in Pterygota, adult moulting became a more dangerous and potentially hazardous event. Consequently, there must have been a high selection pressure towards the suppression of imaginal moulting in the stemgroup of Pterygota. In fact, with rare exemptions, which may be interpreted as neotenic reversals [38], pterygote insects do not moult as adults. The loss of adult moults with a final moult from nymph to a sexually reproductive imago has been regarded as autapomorphic trait of Pterygota [17], although the subimaginal stage in mayflies points to an intermediate position in this respect. The subimago has thus mostly been interpreted as a plesiomorphic remnant of an adult moulting, which became fully suppressed in other pterygotes. Different explanations on the persistence of this penultimate winged life stage in mayflies have been put forward: In subimagines, the dull wing membrane is densely covered with microtrichia and the hind margins of the wings are equipped with marginal setae, both of which lead to an unwettability of the wings [5, 13]. However, the microsculpture of imaginal wings in mayflies also provides hydrophobic properties [14], so unwettability alone cannot be the sole reason for pertaining a separate winged life stage. Maiorana [16] pointed to the significant differences in the lengths of tail filaments and male forelegs between subimago and imago, which are used by the male imagines in a highly specialized aerial mating flight. Males usually aggregate in male mating swarms to perform a characteristic flight pattern, which also includes parachuting manoeuvers aided by the widely spread tail filaments. Males approach incoming females from ventrally, using their extremely elongated forelegs to wrap these around the female forewing bases, thereby anteriorly stabilizing the coupling during copulation [6]. Maiorana [16] assumed that the extremely elongated forelegs and tail filaments in the adult could not be accomplished in a single moult from nymph to imago, hence the necessity to maintain the subimaginal stage. This theory is indirectly supported by the observation that even in those taxa, in which the females remain in the subimaginal stage, the males always retain a subimaginal moulting, by which the extreme elongation of tails and forelegs is accomplished.

In any case, with the necessity to maintain a subimaginal stage, bullae play a crucial role in moulting. They are not only present in extant mayflies, but can also be seen in fossil mayflies of the Cretaceous (Fig. 9A–F), which however all belong to the crowngroup Ephemeroptera [39–41]. The latter differ from Permian stemgroup mayflies like Protereismatidae (Ephemerida: Permoplectoptera) in the posteriorly directed wing pads and the significant reduction of the hind wings [12, 42]. Moreover, growing winglets in nymphs of modern Ephemeroptera are folded and compressed inside short wing pads, which are more or less integrated into the thorax and directed posteriorly, whereas wing anlagen in nymphs of Protereismatidae were unfolded and gradually growing posterolaterad, thus visibly enlarging throughout their nymphal development [12, 42]. The same mode of wing development occurred not only within Ephemerida, but also in other Palaeopterous insect orders (e.g. Palaeodictyopterida, but see [43]). So far there were no details known on the moulting procedure of these gradually outgrowing winglets, nor anything was known about the presence or absence of bullae in the wings of any Paleozoic taxa, being it Ephemerida, Odonatoptera or even Palaeodictyopterida. However, we were able to verify for the very first time well-developed bullae in the wings of Permian Protereismatidae. The holotype of *Protereisma insigne* is an excellently preserved hind wing, which has clearly visible bullae at least on veins Sc, R2, R4 + 5, and MP1. This noteworthy finding suggests that bullae were present throughout Ephemerida and therefore hardens the evidence for the presence of a subimaginal stage at least in stemgroup mayflies. Bullae should be reliable indicators for the presence of adult moultings also in other fossil Palaeopterous groups like Odonatoptera or Palaeodictyopterida (for an overview see [44]), provided that such delicate structures are preserved in pristine condition. Most interesting however is that the bullae were found in a hind wing. This must be due to the fact that, unlike in modern Ephemeroptera, both pairs of wings were of similar size and must have faced the same requirements during moulting. It is thus most probable that Protereismatidae were equipped with bullae in both fore- and hind wings, which aided in the moulting process of these long airfoils.

Before our findings, the two different scenarios for the wing development in Pterygota were equally likely: Either bullae and the subimago were groundplan characters of Pterygota, which were later suppressed in Odonatoptera and Neoptera, or bullae were a novelty of Ephemerida as a consequence of maintaining the subimaginal life stage. With the discovery of bullae already in Paleozoic Ephemerida, it has opened the likeliness that bullae may have been present also in early Odonatoptera, Palaeodictyopterida or even in the stemgroup of Neoptera. The answer to this open question lies in the reinvestigation of well-preserved wings of other Paleozoic taxa in this respect to further unravel the origins of bullae. Fossil bullae are however delicate structures and even mostly ignored in taxonomic descriptions of extant species, let alone in fossil ones. In any case, it would be decisive to learn more about the distribution and evolutionary history of bullae in Pterygota as a key to their early evolution.

## Conclusions

Wing bullae in mayflies are weakened spots in certain longitudinal veins, which are determinant for wing bending during moulting from subimago to imago, but, unlike assumed before, do not play any role in flight. There is a general tendency for reduction of bullae within mayflies. Bullae in mayfly wings can be traced back to Permoplectoptera: Protereismatidae of the Early Permian and are thus assumed to be a groundplan character of Ephemerida. Information on the presence of bullae in other Paleozoic taxa may allow to answer the question if adult moulting was a common trait among early winged insects or an evolutionary novelty developed in the early stemgroup of mayflies.

## Methods

Our studies were based on specimens of extant Ephemeroptera from most of the currently recognized families (Table 1), which are deposited in the collections of the Instituto de Biodiversidad Neotropical (IBN), Tucumán, Argentina and the State Museum of Natural History, Stuttgart, Germany (SMNS).

Investigated fossil specimens of *Borinquena parva* (Leptophlebiidae), *Siphloplecton* sp. (Metretopodidae), and *Burmella paucivenosa* (Vietnamellidae) are deposited in the amber collection of SMNS (see also [39–41]). The undescribed fossil Baetidae in Burmese amber is hosted in the private collection of Patrick Müller, Käshofen, Germany. The undescribed species of Australiphemeridae from Lebanese amber comes from the Dany Azar collection, deposited in the Natural History Museum of the Lebanese University, Faculty of Sciences II, Fanar, Lebanon. The wing photograph of *Protereisma insigne* Tillyard, 1932 (YPM IP 001112) by S. Butts (YPM) is courtesy of the Peabody Museum of Natural History, Division of Vertebrate Paleontology, Yale University, New Haven, CT, USA (http://peabody.yale.edu), 2023. The wing photograph of *Siphluriscus chinensis* Ulmer, 1920 is courtesy of Chang-Fa Zhou, Nanjing Normal University, Nanjing, China.

### Video recording

To video record the process of subimaginal wing moulting and the possible role of bullae therein, we kept subimagines of different species under artificial lighting in transparent acrylic cups. The moulting of subimagines was video recorded with a 2.8/105 mm AF-S Nikkor Micro lens on a Nikon D5300 camera at 60 fps or with a 2.8/105 mm VR-S Nikkor Z lens on a Nikon Z9 camera at 120 fps. In post production, the reproduction speed was partly speeded up during the initial phase of moulting and slowed down at the time of wing bending to show this critical moment in detail. For post production and extraction of still photographs from the videos, Adobe Premiere Pro© software was used.

### High-speed video recording

Imagines were high-speed video recorded at lift-off under artificial conditions in a Hakuba mini studio with a 1.8 / 50 mm AF Nikkor lens on a Fastec TS5 High Speed Digital Camera at varying speed from 900 to 2000 fps. Videos were edited with FasMotion® 2.4.1.

### Cardboard wing models

To test the functionality of the wing membrane and veins, we printed photographed wings on 200 g cardboard paper and cut out the printed wings. The wings were folded along the main longitudinal and intercalary veins to reproduce the actual corrugation of the wing. To model the function of bullae, the paper was pierced by a knitting needle at bulla positions to emulate the respective weakening of the veins.

### Optical microscopy

Wings were observed either directly on specimens in alcohol or wings were dissected and mounted under alcohol gel, glycerine, Euparal, Hydromatrix, or dry on slides. Wing photographs of Neotropical species were taken with a Zeiss Stemi 508 stereomicroscope and a Zeiss AxioScope A1 with an Axiocam ICc5 Camera and processed with Zeiss Zen® software (Jena, Germany). Other extant and fossil species were photographed in series with different focal planes through a Leica Z16 APO Macroscope equipped with a Leica DFC450 Digital Camera using Leica Application Suite v. 3.1.8. Resulting photo stacks were processed with Helicon Focus Pro to obtain combined photographs with extended depth of field. Photographs were sharpened, and contrast and tonality were adjusted using Adobe Photoshop™ version 24.2 (Adobe Systems Incorporated, San Jose, USA).

### Confocal laser scanning microscopy

For confocal laser scanning microscopy (CLSM), wings of ethanol-stored specimens were transferred to glycerol droplets (Electron Microscopy Sciences) on a 1.5 glass coverslip (VWR), with Blu-tack (Bostik™) placed in each corner. A second 1.5 glass coverslip was placed on the Blu-tack and pressed down until the glycerol droplet adhered to the surface of both coverslips [45]. Glass coverslips containing the wings were examined with a Nikon A1R-HD CLSM at the University of New Hampshire Instrumentation Center (Durham, NH, USA). Samples were scanned using three excitation wavelengths: 409.3 nm, 487.8 nm, and 559 nm, and three emission ranges of 400–490 nm, 510–540 nm, and 560–590 nm, respectively. The use of the 409.3-nm laser allowed us to visualize resilin [46]. Scanned image files were rendered and processed using FIJI [47]. Pseudocolours “Blue,” “Green,” and “Red” were applied to channels 1 (400–490 nm), 2 (510–540 nm), and 3 (560–590 nm), respectively.

### Scanning electron microscopy

For scanning electron microscopy (SEM), wings were dissected, subsequently dehydrated through a stepwise immersion in ethanol, dried by critical point drying (Leica EM CPD300), and mounted on SEM stubs. The mounted material was coated with a 5-nm Au/Pd layer (Leica EM ACE200) and subsequently examined and photographed with a Zeiss EVO LS 15 scanning electron microscope. All photographs were subsequently sharpened and adjusted in contrast and tonality in Adobe Photoshop™ version 24.2 (Adobe Systems Incorporated, San Jose, USA).

### Synchrotron X-ray microtomography

For synchrotron X-ray microtomography (µCT), small pieces of ethanol-fixed wings were scanned at the IPS UFO station at KIT Light Source. A parallel polychromatic X-ray beam produced by a 1.5-T bending magnet was spectrally filtered by 0.5 mm aluminum to remove low energy components from the beam. The resulting spectrum had a peak at about 15 keV, and a full-width at half maximum bandwidth of about 10 keV. The samples were scanned at a magnification of 10×, resulting in an effective pixel size of 1.22 µm. We employed a fast indirect detector system consisting of a 13 µm LSO:Tb scintillator [48], a diffraction limited optical microscope (Optique Peter) [49] and a 12 bit pco.dimax S4 high-speed camera with 2016 × 2016 pixels resolution. For each scan, we took 200 dark field images, 200 flat field images and 3000 equiangularly spaced radiographic projections in a range of 180° with 10 ms exposure time each, resulting in a scan duration of 34 s. We used the control system concert [50] for automated data acquisition and online reconstruction of tomographic slices for data quality assurance. The final tomographic 3D reconstruction was performed by tofu [51] and additionally included phase retrieval [52], ring removal, 8-bit conversion and blending of phase and absorption 3D reconstructions in order to increase contrast between the background and homogeneous regions, while at the same time highlighting the edges. Volume renderings of the regions of interest were created with Drishti 2.5.1 [53] (Fig. 6 & Video S6) and Amira 2021.1 (Video S7).

### Supplementary Information

**Additional file 1: Video S1.** High speed videography of *Thraulodes cochunaensis* (Leptophlebiidae) at lift-off (recorded at 1677 frames/seconds, reproduced at 30 frames/second). 

**Additional file 2: Video S2.** High speed videography of *Leptohyphes eximius* (Leptohyphidae) at lift-off (1530 frames/seconds, reproduced at 30 frames/second). 

**Additional file 3: Video S3.** Mating swarm of *Leptohyphes eximius* (Leptohyphidae). 

**Additional file 4: Video S4.** Mating swarm of *Miroculis fittkaui* (Leptophlebiidae).

**Additional file 5: Video S5.** Mating swarm of *Lachlania* sp. (Oligoneuriidae).

**Additional file 6: Video S6.** Subimago of *Ephemera danica* (Ephemeridae), µCT rendering of costa and subcosta at level of bulla sc. 

**Additional file 7: Video S7.** Imago of *Ephemera danica* (Ephemeridae), µCT rendering of subcosta at level of bulla sc.  

**Additional file 8: Video S8.** Moulting of *Chiloporter eatoni* (Ameletopsidae) from subimago to imago. 

**Additional file 9: Video S9.** Moulting of *Callibaetis guttatus* (Baetidae) from subimago to imago.

**Additional file 10: Video S10.** Moulting of *Ephemera danica* (Ephemeridae) from subimago to imago.   

**Additional file 11: Video S11.** Cardboard wing model of *Ephemera danica* (Ephemeridae).

## Data Availability

All data generated or analysed during this study are included in this published article and its supplementary information files.
